# Have your say in the *Eurosurveillance* evaluation survey

**DOI:** 10.2807/1560-7917.ES.2024.29.48.2411289

**Published:** 2024-11-28

**Authors:** Eurosurveillance editorial team

**Affiliations:** 1 European Centre for Disease Prevention and Control (ECDC), Stockholm, Sweden

As a weekly published scientific journal, *Eurosurveillance* is dedicated to robustness, quality and usefulness for its audience. The editorial team is committed to constantly improve the journal’s impact on public health in Europe by providing relevant content for timely public health action and long-term policy-making.

**Tell us how the journal is doing**

We are interested in our readers’ and contributors’ views on the journal that can help us identify our strengths and areas of improvement to inform decisions on the future direction of *Eurosurveillance*. 

The survey is part of an evaluation to assess the journal’s performance in the past 5 years, and to advise how the journal will remain relevant in the future.

Whether you are a contributing author, reviewer, or reader of *Eurosurveillance*: we value your opinion and would love to hear about your experience. 

Taking the survey will take around 15–20 minutes.

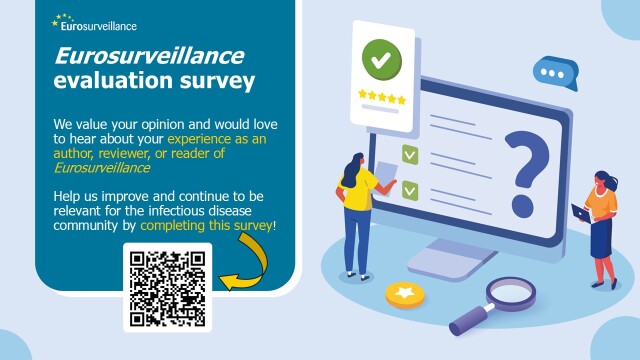



Tell us what you think and participate in our survey and let us know how we are doing: access the survey here (please feel free to also share the link with others).

